# A longitudinal study of the individual‐ and group‐level problematic gaming and associations with problem gambling among Swedish adolescents

**DOI:** 10.1002/brb3.949

**Published:** 2018-03-12

**Authors:** Sofia Vadlin, Cecilia Åslund, Kent W. Nilsson

**Affiliations:** ^1^ Centre for Clinical Research Uppsala University Västmanland County Hospital Västerås Sweden

**Keywords:** Adolescence, behavioral addiction, comorbidity, gambling problems, gaming problems

## Abstract

**Aim:**

The aims of this study were to investigate the long‐term stability of problematic gaming among adolescents and whether problematic gaming at wave 1 (W1) was associated with problem gambling at wave 2 (W2), three years later.

**Methods:**

Data from the SALVe cohort, including adolescents in Västmanland born in 1997 and 1999, were accessed and analyzed in two waves W2, *N *=* *1576; 914 (58%) girls). At W1, the adolescents were 13 and 15 years old, and at W2, they were 16 and 18 years old. Adolescents self‐rated on the Gaming Addiction Identification Test (GAIT), Problem Gambling Severity Index (PGSI), and gambling frequencies. Stability of gaming was determined using Gamma correlation, Spearman's rho, and McNemar. Logistic regression analysis and general linear model (GLM) analysis were performed and adjusted for sex, age, and ethnicity, frequency of gambling activities and gaming time at W1, with PGSI as the dependent variable, and GAIT as the independent variable, to investigate associations between problematic gaming and problem gambling.

**Results:**

Problematic gaming was relative stable over time, γ = 0.739, *p *≤* *.001, ρ = 0.555, *p *≤* *.001, and McNemar *p *≤* *.001. Furthermore, problematic gaming at W1 increased the probability of having problem gambling three years later, logistic regression OR = 1.886 (95% CI 1.125–3.161), *p *=* *.016, GLM *F* = 10.588, η^2^ = 0.007, *p = *.001.

**Conclusions:**

Problematic gaming seems to be relatively stable over time. Although associations between problematic gaming and later problem gambling were found, the low explained variance indicates that problematic gaming in an unlikely predictor for problem gambling within this sample.

## 1 INTRODUCTION

Studies on problematic gaming among adolescents have rapidly increased in recent years (Desai, Krishnan‐Sarin, Cavallo, & Potenza, 2010; Kuss & Griffiths, 2012a,b; Lemmens, Valkenburg, & Peter, 2009; Rehbein & Baier, 2013). However, longitudinal studies are scarce, and the existing studies on the stability and trajectories of problematic gaming are ambiguous (Gentile et al., 2011; King, Delfabbro, & Griffiths, 2013; Konkolÿ Thege, Woodin, Hodgins, & Williams, 2015; Liau et al., 2014; Scharkow, Festl, & Quandt, 2014; Van Rooij, Schoenmakers, Vermulst, Van Den Eijnden, & Van De Mheen, 2011). In 2013, when Internet gaming disorder (IGD) was included in the DSM‐5, section 3 “Conditions for further studies” (American Psychiatric Association, 2013), it was stated that several aspects needed to be evaluated further before considering IGD as an actual diagnosis. One of those aspects is the natural course. A two‐year longitudinal study of children in Singapore found that problematic gaming was persistent and not solely a symptom of comorbid disorders (Gentile et al., 2011; Liau et al., 2014). Similarly, a study of adolescents in the Netherlands reported that half of the addicted gamers were still addicted one year later (Van Rooij et al., 2011). In contrast, a German study of adolescents and adults indicated that only 1% of the problematic gamers were still problematic gamers one year later (Scharkow et al., 2014). A three‐wave panel of adult gamers showed a decline in problematic gaming over time, although self‐reported problematic gamers scored higher on problematic gaming tests at all three time points (King et al., 2013). A five‐year longitudinal study of adults in Canada showed that problematic gaming was fairly transient, as were most of the investigated behavioral addictions (Konkolÿ Thege et al., 2015).

Similarities between problematic gaming and problem gambling have been described previously and include both potential biological and biochemical similarities, as well as structural characteristics such as the variable ratio of intermittent reinforcement schedules and the use of sound, light, and graphic effects (Grant, Brewer, & Potenza, 2006; Grant, Potenza, Weinstein, & Gorelick, 2010; Griffiths, 2005; Griffiths & King, 2015; Griffiths & Parke, 2010; Kuhn et al., 2011; Kuss & Griffiths, 2012a; Leeman & Potenza, 2012; Leeman & Potenza, 2013; Pontes & Griffiths, 2014). In recent years, the increasingly indistinct boundaries between digital games regarding bonds and prizes have increasingly blurred the distinction between gaming and gambling activities (Griffiths & King, 2015). So‐called social‐games that can be played free of charge (or for real money if buying extra spins, bonds, etc.) are common in social media settings, and in a 2008 study of adolescents in Oregon, it was found that free gambling was the most popular online activity, whereas only a few adolescents had gambled for real money online (Volberg, Hedberg, & Moore, 2008). Additionally, a study of adult social gamers who never gambled online showed that approximately 26% had migrated to online gambling six months later (Kim, Wohl, Salmon, Gupta, & Derevensky, 2015), and in a study of adult social casino gamers with self‐reported gambling problems, 10% reported problematic use of social casino games despite the lack of financial incentives to play (Gainsbury, King, Russell, Delfabbro, & Hing, 2017).

Studies on associations between problematic gaming and problem gambling/gambling disorder so far have given ambiguous results ( Delfabbro, King, Lambos, & Puglies, 2009 Fu & Yu, 2015; King, Ejova, & Delfabbro, 2012; Wood, Gupta, Derevensky, & Griffiths, 2004). In a study of adolescents in Australia, it was concluded that although the frequency of video‐game playing was significantly related to pathological gambling, the effect size was very small, and that it was unlikely that video gaming was a significant risk factor for pathological gambling (Delfabbro, Winefield, & Anderson, 2009). In another Australian study of adult gamers and adult gamblers, gaming in itself was not associated with gambling, although those who both gambled and gamed had similar perceptions of direct control over chance‐based gambling events (King et al., 2012). Contrary to this, in a study of Canadian children and adolescents, a clear relationship was found between video‐game playing and gambling, and problem gamblers were significantly more likely to spend excessive time gaming than those who did not gamble (Wood et al., 2004). Moreover, a study of Chinese adolescents and young adults found that being classified as an Internet gaming addict was a significant risk factor for disordered gambling and that the severity of Internet gaming addiction positively varied with the severity of disordered gambling, even though the effect size was small (Fu & Yu, 2015). In a recent systematic review of problematic gambling among adolescents in Europe (Calado, Alexandre, & Griffiths, 2017), prevalence of 0.2–12% was indicated. Although gambling is illegal for those under the age of 18 years in Sweden, it is still considered to be common among youths, and in 2008/2009, 3.5% of 16–17 year olds were estimated to have gambling problems (Folkhälsomyndigheten, 2010). This number is similar to estimates among youths in European countries, North America, and Oceania (Volberg, Gupta, Griffiths, Ólason, & Delfabbro, 2010), and it was concluded that problem gambling was highly transitional in nature among adolescents. A two‐wave study of 16–24‐year olds in Sweden suggested there was a high degree of mobility in and out of gambling problems over time on an individual level (Fröberg et al., 2015). Recovery from gambling problems was high, particularly among females, and transitions between problem and nonproblem gambling were common (Fröberg et al., 2015).

The aims of this study were to investigate the stability of problematic gaming and associations with problem gambling three years later.

## 2 METHODS

### 2.1 Participants

#### 2.1.1 First wave (W1)

**Figure 1:**
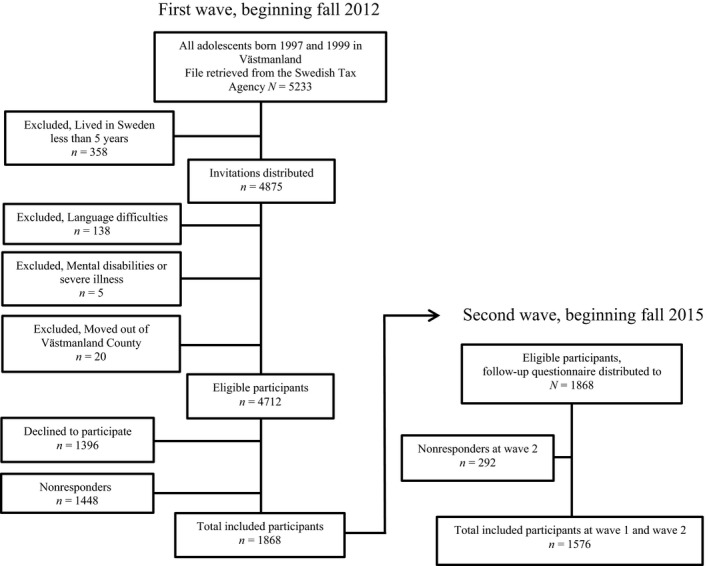
Flowchart of the study population

Adolescents born in 1997 and 1999 and living in the county of Västmanland in Sweden were included in a prospective cohort study (the Survey of Adolescent Life in Västmanland, SALVe cohort) starting in the fall of 2012 (Vadlin, Åslund, Rehn, & Nilsson, 2015). The adolescents were contacted by regular mail and asked to participate in the study by completing a self‐report questionnaire. The total study population consisted of 1868 adolescents (1035 girls, 55.4%). Among the participants, 945 (50.6%) were born in 1997, and 392 (21%) were classified as being of non‐Scandinavian ethnicity. The total response rate was 40% (Figure 1). Analysis presented in this study, however, only includes adolescents included at both waves (*n *= 1576).

#### 2.1.2 Second wave (W2)

In the second wave, starting in the fall of 2015, the adolescents were once again contacted by regular mail and asked to answer a second self‐report questionnaire, similar to the one in the first wave. The total study population at W2 consisted of 1576 adolescents (914 girls, 58%). Among those, 797 (50.6%) were born in 1997, and 314 (20%) were classified as being of non‐Scandinavian ethnicity. The total response rate was 84% (Figure 1).

### 2.2 Ethics

The study was approved by the Ethical Review Board in Uppsala, Dnr: 2012/187, and in accordance with the Declaration of Helsinki. All the adolescents and their parents gave written informed consent to participate in the study.

### 2.3 Measurements

*The Gaming Addiction Identification Test (GAIT)* is a screening instrument for symptoms of gaming addiction in adolescents within the last 12 months (Vadlin, Åslund, & Nilsson, 2015). The GAIT originally consisted of 15 items on a five‐point scale ranging from 0 =  “*disagree*” to 4 =  “*completely agree,*” with a possible total of 52 points, because the first two items are not included in the scoring (Vadlin, Åslund, et al., 2015). The 15‐item version of GAIT has been reported to have high internal consistency (α = 0.906), high concordance in adolescent–parent ratings (ρ = 0.704), and high concurrent validity (ρ = 0.834) (Vadlin, Åslund, et al., 2015) with the 7‐item version of the Gaming Addiction Scale for Adolescents (GAS) (Lemmens et al., 2009). In order to use the GAIT in further studies and to be able to compare it with other IGD‐based scales, two additional questions were added, one regarding lying/deception and one regarding escape/mood modification (Vadlin, Åslund, et al., 2015). The new 17‐item version of the GAIT, covering all nine IGD‐criteria, was included in W2. However, to be able to compare the first and second waves, the 15‐item version was used in the analyses of this study. Internal consistency for the GAIT scale measured by Cronbach's alpha was α = 0.901 (95% CI 0.893–0.908) in W1 and α = 0.891 (95% CI 0.883–0.899) in W2. Due to the severe zero inflation, GAIT was divided into quartiles in the logistic regression, where Q1 had lowest scores and Q4 the highest, and Q1 was set as reference category. Q1 = 0p (29.2%), Q2 = 1–3p (21.7%9, Q3 = 4‐10p (25.6%), and Q4 ≥ 11p (23.6%). In this study, individuals belonging to Q4 division of the GAIT are referred to as problematic gamers.

*The Problem Gambling Severity Index (PGSI)* is a nine‐item self‐rating scale developed to measure symptoms of problem gambling within the last 12 months, in general populations (Ferris & Wynne, 2001), and was used in W2. It has response options of 0–3 (“*never”* to *“almost always”*) and a total of 27 points. In this study, a cutoff of ≥3 points was set as an indication of problem gambling, as commonly used and previously suggested by the Public Health Agency of Sweden (Folkhälsomyndigheten, 2010). Internal consistency for the PGSI measured by Cronbach's alpha was α = 0.698 (95% CI 0.651–0.741) in W2.

*Gambling activities* at W1 were measured by four questions regarding the frequency of gambling activities during the last 12 months: 1) online casino or poker, 2) offline casino or poker, 3) offline slot machines, and 4) sports betting. All questions had seven response options ranging from never to 5–7 days a week (*0 =  Never, 1 =  A few times a year, 2 =  A few times a month, 3 = 2–4 times a month, 4 = 2–3 days a week, 5 = 4–5 days a week, and 6 = 6–7 days a week*).

*Frequency and duration of gaming activities* at W1 were measured by three questions regarding frequency and duration of gaming activities on week days and on weekends divided by level of violent content. Response options ranging from never to 6–7 days a week (*0 =  Never, 1 =  A few times a year, 2 =  A few times a month, 3 = 2–4 times a month, 4 = 2–3 days a week, 5 = 4–5 days a week, and 6 = 6–7 days a week*) for frequency, and response options for duration ranging from 0 to more than 5 h (on a VAS‐scale with 30‐minute intervals). An index was made by computing frequency and duration on a total week regardless of degree of violent content.

#### 2.3.1 Control variables

*Age* was coded as year of birth (1997 and 1999).

*Sex* was coded as girls = 0, boys = 1.

*Ethnicity* was coded as 0 = Scandinavian, 1 = Non‐Scandinavian. Participants whose parents were both born in Sweden or Scandinavia were classified as Scandinavian, while those with at least one parent born outside Scandinavia were coded as non‐Scandinavian.

### 2.4 Statistical analysis

Percentages were calculated for descriptive statistics, and chi‐square tests were performed to analyze dichotomous variables and sex differences, and t test for analyzing sex differences in continuous variables. Cronbach's alpha was computed to measure the internal consistency of the GAIT at both waves, and for PGSI. Spearman's rho (ρ) was used to investigate correlations between GAIT at W1 and W2 on scale level; Gamma correlation (γ) was used to investigate correlations between the dichotomized quartile divided versions of the GAIT (GAIT Q1‐Q3 and Q4), and the McNemar test was performed to analyze differences between changes in problematic gaming between the two time points. Because the data were positively skewed and zero‐inflated, logistic regression analysis was performed using problematic gaming measured by the quartile divided version of GAIT, frequency of gaming and gambling activities, and adjusted for sex, age, and ethnicity to predict problem gambling measured by the dichotomized version of the PGSI, with ≥ 3 points as the cutoff. Additional general linear model (GLM) analysis was performed for problematic gaming measured by GAIT and frequency of gaming and gambling activities, and adjusted for sex, age, and ethnicity to predict problem gambling measured by PGSI, to validate the results from the logistic regression analysis. All described analyses were performed using the Statistical Package for Social Sciences (version 24; IBM SPSS, Armonk, NY). Statistical significance was set at *p *<* *.05.

## 3 RESULTS

### 3.1 Dropout of gamers between W1 and W2

Although the response rate was high (84%) at W2, 20.4% of the problematic gamers dropped out at W1, compared to 14.4% of nonproblematic gamers, and among the dropouts 21% were boys compared to 12% of the girls (data not shown). In the dropout group, 20.3% were non‐Scandinavian compared with 14.9% Scandinavian (*p *=* *.009), and 23.2% of those who gamed more than 30 h/w dropped out compared with 13.8% of those who played <30 hr/w (*p *≤* *.001). None of those who participated in gambling activities more than twice a month dropped out; however, there were too few participants to be able to perform a statistical analysis (data not shown).

### 3.2 Descriptive features of the W1 and W2 participants

Distributions of age and ethnicity were similar in both sexes (Table 1). Problematic gaming was approximately four times more common among boys than girls at W1, compared to approximately five times more common among boys at W2. Problem gambling was almost eight times more common among boys. The frequency of gambling activities was low in both sexes, although higher among boys compared to girls; however, only frequency of sports betting presented a significant result. Of all adolescents at W1, 0.3% had played online casino/poker two times a month or more, 0.1% had played offline casino/poker two times a month or more, 0.1% had played offline slot machines two times a month or more, and 0.6% had participated in sports betting two times a month or more, all within a 12‐month period (Table 1).

**Table 1:** Descriptive statistics for measurements in first and second wave of the SALVe Cohort

	Total	Boys	Girls	Sex differences	
	*n* (%)	*n* (%)	*n* (%)	*Χ* ^2^	*p‐*value
Age					
1997	797 (50.6)	340 (42.7)	457 (57.3)	0.284	.594
1999	779 (49.4)	322 (41.3)	457 (58.7)		
Ethnicity					
Scandinavian	1258 (80)	527 (41.9)	731 (58.1)	0.002	.963
Non‐Scandinavian	314 (20)	132 (42.0)	182 (58.0)		
GAIT wave 1					
Nonproblematic gamer, Q1–Q3	1175 (76.4)	381 (32.4)	794 (67.6)	204.035	<.001
Problematic gamer, Q4	362 (23.6)	271 (74.9)	91 (25.1)		
GAIT wave 2					
Nonproblematic gamer, Q1–Q3	1227 (80.3)	409 (33.3)	818 (66.7)	228.060	<.001
Problematic gamer, Q4	301 (19.7)	245 (81.4)	56 (18.6)		
PGSI wave 2					
Nonproblematic gambling 0–2p	1510 (98.3)	634 (42.0)	876 (58.0)	22.554	<.001
Problematic gambling ≥ 3p	26 (1.7)	23 (88.5)	3 (11.5)		
Gaming frequency last 12 months, wave 1					
<30 hr/w	1182 (80.8)	357 (58.0)	825 (97.4)	357.632	<.001
30 hr/w or more	281 (19.2)	259 (42.0)	22 (2.6)		
Gaming frequency last 12 months, wave 1 (total scale)				*t*	
Total gaming time per week, *n*	1470 (100)	622 (42.3)	848 (57.7)	30.118	<.001
Total gaming time per week, mean (SD)	14.900 (19.978)	29.311 (21.916)	4.329 (8.701)		
Total gaming time per week, range	0–115.500	0–115.500	0–66.000		
Gambling frequency last 12 months, wave 1				*Χ* ^2^	
Online casino, poker					
Never to a few times a month	1537 (99.7)	648 (42.2)	889 (57.8)	1.763	.184
≥2 times a month to daily	4 (0.3)	3 (75.5)	1 (25.0)		
Offline casino, poker					
Never to a few times a month	1562 (99.9)	657 (42.1)	905 (57.9)	2.750	.097
≥2 times a month to daily	2 (0.1)	2 (100)	‐		
Offline slot machines					
Never to a few times a month	1559 (99.9)	655 (99.7)	904 (100)	2.755	0.097
≥2 times a month to daily	2 (0.1)	2 (0.3)	‐		
Sports betting					
Never to a few times a month	1541 (99.4)	648 (98.8)	893 (99.8)	5.863	.015
≥2 times a month to daily	10 (0.6)	8 (1.2)	2 (0.2)		

### 3.3 Stability of problematic gaming

The group stability of self‐rated problematic gaming was analyzed with Spearman's rho for the GAIT scale, ρ = 0.555, *p *≤* *.001, and the individual stability analyzed with Gamma correlation for problematic gaming using the quartile divided GAIT, γ = 0.739, *p *≤* *.001 (Table 2). Of the nonproblematic gamers at W1, 88.6% reported no problematic gaming at W2, and 11.4% reported problematic gaming at W2. Of the problematic gamers at W1, 53.9% had no problematic gaming at W2, and 46.1% were still problematic gamers. The proportion of discordant pairs was 27.6%. Stability on group level indicated a small reduction in problematic gaming between W1 and W2 (*p *≤* *.001) (not shown in table). Furthermore, the McNemar test showed a change between the two time points in problematic gaming, *p *≤* *.001. Moreover, as seen in Table 2, 360 adolescents were problematic gamers at W1. When analyzed separately, no differences were found between problematic and nonproblematic gamers (χ^2^ = 2.317, *p *=* *.128).

**Table 2:** Individual stability of problematic gaming, wave 1 and wave 2

	No problematic gaming W2, *n* (%)	Problematic gaming W2, *n* (%)	Total, *n* (%)
No problematic gaming W1	1003 (88.6)	129 (11.4)	1132 (75.9)
Problematic gaming W1	194 (53.9)	166 (46.1)	360 (24.1)
	1197 (80.2)	295 (19.8)	1492 (100)

Gamma correlation γ = 0.739, *p *= <.001.

Correlation between GAIT scale and frequency of gaming time at W1 was slightly higher, (ρ = 0.641, *p *≤* *.001), compared to W2 (ρ = 0.599, *p *≤* *.001) (not shown in table).

### 3.4 Association between problematic gaming at W1 and problem gambling at W2

As there was a high zero inflation in the PGSI as in GAIT, the statistical power was low in the analysis; hence, both a logistic regression analysis and a GLM analysis were performed.

Table 3 presents the prediction of problem gambling at W2 by problematic gaming at W1, and frequencies of gaming and gambling activities at W1, adjusted for sex, age, and ethnicity. Problematic gaming at W1, male sex, younger age, and offline poker or casino activities was the only significant variables in the GLM for predicting problem gambling at W2, and the model explained approximately 3.7% of the variation in problem gambling. The explained variance for problematic gaming alone was η^2^ = 0.7% of the 3.7% explained variance in the total model.

**Table 3:** General linear model of total problematic gaming measured by GAIT at W1, frequency of gaming and gambling activities at W1 predicting problematic gambling at W2. Multivariable logistic regression of GAIT at W1, frequency of gaming and gambling activities at W1, predicting problematic gambling at W2

	*F*	*p*	η^2^	OR (95% CI)	*p*
GAIT^a^,^b^	10.588	.001	0.007	1.886 (1.125–3.161)	.016
Male sex	11.852	.001	0.008	0.201 (0.055–0.733)	.015
Age (increasing)	10.467	.001	0.007	0.332 (0.130–0.846)	.021 ns
Non‐Scandinavian ethnicity	–	ns	–	–	
Gaming time per week	–	ns	–	–	ns
Online poker, or casino	–	ns	–	–	ns
Offline poker, or casino	7.299	.007	0.005	–	ns
Offline slot machines	ns	ns	–	–	ns
Sports betting	–	ns	–	–	ns
	Adj. *R* ^2^ = 0.037			Nagelkerke *R* ^2^ = 0.147	

a GAIT scale in GLM.

b GAIT quartiles in logistic regression

In the logistic regression analysis, adolescents with problematic gaming at W1 had an almost two times greater probability of problem gambling at W2 (Table 3). In line with the GLM, also male sex, and younger age was significantly associated with problem gambling, whereas offline poker or casino activities were nonsignificant. Similar to the GLM, none of the other variables in the model presented a significant association with problem gambling. The total model explained 14.7% of the variation in problem gambling. In a univariate binary logistic regression, using only problematic gaming at W1 as predictor of problem gambling at W2, the probability was slightly higher, OR = 2.503 (95% CI 1.548–4.046), *p ≤ *.001, although a lower explained variance at 8.3% (not shown in table).

A positive association was found between higher quartile division of the GAIT and problem gambling by PGSI (Q1 = 0% problem gambling, Q2 = 3.8%, Q3 = 8.0%, and Q4 = 12.1% problem gambling). Correlation analysis between PGSI scale and frequency of gaming time was ρ = 0.245, *p *≤* *.001. Correlations between PGSI scale and frequency of gambling activities at W1 for online casino, poker were ρ = 0.036, ns, offline casino, poker ρ = 0.143, *p *≤* *.001, slot machines ρ = 0.146, *p *≤* *.001 and sports betting ρ = 0.036, ns.

## 4 DISCUSSION

The present study aimed to investigate the stability of problematic gaming, and associations between problematic gaming and later problem gambling among a cohort of Swedish adolescents.

The self‐rated prevalence of problematic gaming was slightly lower at W2 for the whole adolescent group, as well as when separated by sex. Problem gambling had a high sex difference with a ratio of almost eight boys to one girl. Adolescents in the present study had lower rate of problem gambling: 1.7% compared with 3.5% in a previous study on Swedish adolescents and younger adults (Fröberg et al., 2015), although the sex differences were similar in both studies. At W1, in 2012, the adolescents in the SALVe cohort were 13 and 15 years old, and all gambling activities were illegal for them at that point. At W2, it was still illegal for the adolescents born in 1999 to gamble. The data from the adolescent/adult study (Fröberg et al., 2015) were collected in 2008/2009 and 2009/2010, and a majority of the participants could legally gamble at both waves, which might be one explanation of the lower prevalence of problem gambling in the SALVe cohort. The different time intervals, as well as the age difference between the SALVe cohort and the Swedish adolescent/adult study, might be important; as the adolescents in the SALVe cohort were at risk of developing problem gambling between W1 and W2, whereas some of the participants in the adult/adolescent study may have been in recovery from gambling problems at the time of measuring, as transitions were common (Fröberg et al., 2015). Differences in age might also explain the differences in occurrence between the studies at the time of the second measurement. Furthermore, there was higher proportion of girls than boys in the SALVe cohort as opposed to the Swedish adolescent/adult study, where boys in both studies gamble more and report more problem gambling (Fröberg et al., 2015).

Most of the adolescents in the present study had no problematic gaming at either wave. However, among the problematic gamers at W1, almost half of them also had problematic gaming at W2, and among the problematic gamers at W2, six of 10 also have had previously problematic gaming at W1. This means that 50% of the problematic gamers had recovered at W2. Interestingly, the overall proportion of problematic gamers decreased, most probably as a function of age. In the analysis, all adolescents were included, not just those with problematic gaming at W1. This was to ensure that we detected the trajectories of those who went from nonproblematic gaming at W1 to having developed problems at W2. The probability of having problematic gaming at W2 is not equal between those who had problematic gaming at W1 compared with those without problematic gaming at W1. This interdependency suggests that the use of Gamma correlations was more appropriate than tests focusing on proportional differences or linearity. However, developing problematic gaming is less likely among those who have no problem with gaming. The high Gamma correlation indicates individual stability, in both nonproblematic‐ and problematic gaming, at both waves. Even though the Spearman's rho was considered to be moderate, and the McNemar showed a significant difference between W1 and W2; however, we argue that problematic gaming is relatively stable. Spearman's rho only evaluates the linear relationship between rank‐ordered variables at both waves, and McNemar's test assumes independence among the paired response, were the odds for the outcome at W2 are unequal depending on the state at W1. On the other hand, the Gamma correlation takes into account the fact that there is a dependency and curve‐linearity between problematic and nonproblematic gaming at the two waves. This demonstrates that for those with problematic gaming at W1, there was a higher risk of having problematic gaming at W2, and the phenomenon of problematic gaming is therefore considered to be relatively stable. Moreover, regression toward the mean must be considered (Bland & Altman, 1994), where high/low, or extreme values tend to be closer to the mean on the second measurement. Therefore, regression to the mean further decreases the proportion of stable cases, as those that were miss‐classified as problematic gamers at W1 probably have been classified as nonproblematic gamers at W2. Furthermore, the stability of problematic gaming in the present study lies closer to the results from the children and adolescent studies (Gentile et al., 2011; Liau et al., 2014; Van Rooij et al., 2011) indicating that problematic gaming seems to be a persistent problem, in contrast to the studies of adults that showed problematic gaming to being fairly transient (King et al., 2013; Konkolÿ Thege et al., 2015; Scharkow et al., 2014).

The low correlation between gaming time and GAIT score is in line with previous research that frequency and duration per se are not linearly associated with problematic gaming. Instead it seems probable that other mechanisms are relevant for development of problematic gaming, and this needs to be further explored. Surprisingly, frequency of gambling activities and PGSI scores indicated negligible correlations, where two of three also were nonsignificant. Although an association between problematic gaming and a higher probability of later problem gambling was found both in the logistic regression analysis and in the GLM, the explained variance was low. The results of associations between problematic gaming and later problem gambling in the present study lie at an intermediate point between previous findings in Canada and China that showed a clear relationship between problematic gaming and problem gambling (Fu & Yu, 2015; Wood et al., 2004) compared to the Australian studies that found the associations to be unlikely (Delfabbro, King, et al., 2009; King et al., 2012).

The associations between problematic gaming and problem gambling need further investigations in larger samples, in different countries, among different age groups, and between sexes. Furthermore, additional studies of the stability of gaming and gambling problems among adolescents and adults are needed.

### 4.1 Limitations and strengths

The present study has several limitations. First, only self‐rated symptoms were included in the study; however, the same measurements were used at both waves. Second, there were a higher amount of problematic gamers in the dropout group which may have influenced the results. Third, data on specific gaming activities were not included, data which otherwise might have given information on possible differences between gaming genre and problematic gambling as social casino games have been seen to be associated with problem gambling, Fourth, the low frequency of gambling activities and problem gamblers caused a highly skewed and zero‐inflated data limiting possible of analytical methods; however, both logistic regression analysis and GLM were performed to complement each other. Fifth, stability has been used in the present paper to describe the persistence of problematic gaming; however, stability is commonly used to describe analysis using three or more time points. Sixth, the low internal consistency in PGSI is problematic and might be explained by the fact that there has not been a validation of PGSI in Swedish, or among Swedish adolescents; hence, we do not know how this will affect their interpretation of the questions. Seventh, as there were no data at W1 for problem gambling measured by PGSI, we cannot know if the problem gamblers at W2 would also have been problem gamblers at W1. However, as they were only 13 and 15 years old at W1, we believe that this would probably not be the case. The low gambling activities indicate that most of the adolescents did not participate in any gambling activity at W1 (99.4–99.9% never gambled or gambled <2 times a month). Nevertheless, as there are no data, we can only speculate. Eighth, the arbitrary cutoff used to define problematic gaming in the present study need to be considered; hence, the usage of quartiles is sample depended and therefore affect the results. Lastly, problematic gaming at W2 was not included in the final analysis because the purpose was to investigate associations of earlier problematic gaming in predicting later problem gambling; thus, the inclusion of problematic gaming at W2 would yield ambiguous interpretations of the explained variance of the models.

The study also has strengths. First, the longitudinal design and the use of the same measures enabled comparisons at group and individual levels between the two time periods. Second, this is the first study of adolescents in Sweden to measure the long‐term stability of problematic gaming and associations with problem gambling. Although the occurrence of problem gambling was low, the present study provides insight into the self‐rated prevalence of problematic gaming, problem gambling, and gambling activities among adolescents. Third, the GLM and the logistic regression analysis included control variables, which took into account some of the known possible confounders, although the models explained about 3.7–14.7% of the probability of later problem gambling.

## 5 CONCLUSION

The findings in the present study indicate that problematic gaming seems to be relatively stable over time. The occurrence of gambling activities and problem gambling was low in the sample, although higher among boys. Furthermore, even if associations between problematic gaming and later problem gambling were found, the low explained variance indicates that problematic gaming in an unlikely predictor for problem gambling within this sample.

## AUTHOR CONTRIBUTION

The sponsors of the study had no role in the study design, data collection, data analysis, data interpretation, or writing of the report.

## CONFLICT OF INTEREST

The authors also declare no conflict of interest with organizations that seek to provide help with or promote recovery from addiction.
